# Benefits and barriers in the design of harmonized access agreements for international data sharing

**DOI:** 10.1038/s41597-019-0310-4

**Published:** 2019-12-02

**Authors:** Katie M. Saulnier, David Bujold, Stephanie O. M. Dyke, Charles Dupras, Stephan Beck, Guillaume Bourque, Yann Joly

**Affiliations:** 1 Centre of Genomics and Policy, McGill University, Montreal, Canada; 2 McGill University and Genome Quebec Innovation Centre, Montreal, Canada; 3 McGill Centre for Integrative Neuroscience, Montreal Neurological Institute, McGill University, Montreal, Canada; 4 University College London, London, United Kingdom

**Keywords:** Medical research, Policy, Databases

## Abstract

In the past decade, there has been a surge in the number of sensitive human genomic and health datasets available to researchers via Data Access Agreements (DAAs) and managed by Data Access Committees (DACs). As this form of sharing increases, so do the challenges of achieving a reasonable level of data protection, particularly in the context of international data sharing. Here, we consider how excessive variation across DAAs can hinder these goals, and suggest a core set of clauses that could prove useful in future attempts to harmonize data governance.

DAAs are agreements between data producers and data users that set the terms of use for data sharing. DAAs are an important tool for DACs in providing fair, efficient, and safe access to participant data. The complexity of this task, coupled with a lack of coordination between DACs, has resulted in an overall lack of harmonization in the processes and requirements for data access across projects^1–4^. The fostering of harmonization between databases where appropriate is recognized as a potential solution across a number of consensus documents, including those from the Organisation for Economic Co-operation and Development (OECD), the Human Genome Organisation (HUGO), and the Global Alliance for Genomics & Health (GA4GH)^5–7^. Nevertheless, it has proven difficult to reach consensus on a set of terms and conditions that could be used, with minimal adjustments, by multiple DACs in different jurisdictions^8^. Indeed, research from GA4GH into the challenges of harmonization indicates that a lack of international consensus on data-sharing norms is a significant barrier to collaborations and sharing across borders^9^.

These variations in DAAs exist even across DACs with shared goals and similar types of data. The International Human Epigenome Consortium (IHEC), an international consortium providing “free access to high-resolution reference human epigenome maps for normal and disease cell types to the research community” (http://ihec-epigenomes.org/about/), uses a single portal to provide completely open access to much of its non-individually identifying data. Sensitive data, such as raw sequencing datasets generated by sequencers (FASTQ, BAM or CRAM files), are held within the European Genome-Phenome Archive (EGA) or the Database of Genotypes and Phenotypes (dbGaP) and made available through the DACs of each data-providing member. Each participating project stipulates the terms and conditions of access to its own controlled data, and these terms may be influenced by institutional norms, the project’s national data sharing context, and past experiences and insights from the individuals involved in the drafting.

With the intent of undertaking an internal global quality control analysis of IHEC data – the EpiMAP project – we attempted to develop a harmonized agreement that would be suitable for all IHEC controlled access datasets by examining the DAAs of our own consortium members (seven in total: The BC Cancer Agency, Blueprint, CREST, DEEP Data, the Korea Epigenome Consortium, the McGill Epigenome Mapping Centre, and the Singapore Epigenome Project.) (Both the final harmonized agreement and the seven DAAs examined for its development are available at figshare)^10^. (See Fig. 1). What we discovered was that even *within* a consortium with a shared scientific mission and a commitment to open data sharing, there was significant variation in the content of DAAs. Additionally, many DAAs contained clauses that were lengthy and complex beyond what was needed to communicate the terms of agreement at the expense of clarity. Our work and discussions with colleagues participating in other consortia confirm that the challenge faced by IHEC is not an isolated phenomenon. While there are a number of different governance processes that occur along the way from data collection to data sharing, there is currently no overarching governance of DACs to oversee the corresponding DAAs, which form the bedrock of the relationship between data providers and data users (and their institutions), and as such were our focus here in facilitating a smoother and more streamlined access process. An evaluation of DAC governance is currently underway as part of EU-STANDS4PM. Further below, we present the results of our own internal analysis of these documents, followed by recommendations on how DAA clauses could be employed to facilitate international sharing, based on existing literature on data sharing best practices, as well as on the principle that more streamlined, readable agreements will allow for better comprehension and compliance by data users^1,4–7,11^.

Harmonization practices do not only create benefits for ease of use; they may also facilitate better compliance from researchers in an area where legal mechanisms of enforcement are not always clear. For efficiency reasons, most DAAs are contracts of adhesion, whereby the contract is drawn up exclusively by one party, leaving little room for negotiation of terms. While ubiquitous, contracts of adhesion raise particular issues for compliance^12^; for example, opaque terms and conditions may be unenforceable in court^13^. The practice of having both the researcher *and* a legal representative of their research institution sign a DAA is intended to reduce this particular concern but will not eliminate it if the meaning of the clause remains opaque to the researcher. Meanwhile, references to external conditions or guidance, particularly if a copy of the text does not form a part of the agreement, are rarely enforceable in the context of these types of contracts. Many people will sign such a contract without having closely read its contents, if they read them at all^14^. In the context of research, DAAs may even be viewed merely as an administrative hurdle, to be passed off to an assistant or student to address.

In practice, the normative strength of contractual terms does not rest entirely on the content of the clauses themselves. The relationship between the parties and a sense of “fairness” about the contract can promote compliance more than legal jargon, and research into contractual compliance has shown that a lack of participation in the drafting of a contract can decrease the signer’s commitment to their contractual promise^14^. When the adhering party (in this case, the researcher) perceives the contract to be merely “transactional”– that is to say, when they view the contract as a one-time exchange “devoid of loyalty or commitment” - they are more likely to see their need to comply as malleable^14^. There is value in framing these documents as agreements between parties with mutual interests (i.e. open science and protection of participant privacy) rather than as a straightforward legal transaction, as this may encourage a relationship that creates a larger sense of obligation to the DAA beyond fear of sanctions. While attention has been given to the possibility of legal sanctions to enforce compliance, whether civil or criminal, the availability of effective extrajudicial sanctions (such as the denial of continued access to data for research, retraction of journal publications, or reporting bad behaviour to ethics committees, institutions, employers, or funders)^13^, alongside the high financial costs of pursuing legal remedies, means that legal compliance mechanisms may be neither accessible nor effective.

The contract “as experienced” carries as much or more import than the contract “as written”, as evidenced by the difficulty in enforcing contracts of adhesion^14^. The framing of the contract – its social, rather than legal context – greatly impacts the degree to which its signatories interpret their consents as creating binding obligations^12^. Demands for enforcement framed in *moral* terms – a reminder that the contract is a promise, an obligation that the researchers have created *for themselves* – has the potential to encourage greater compliance^12^. Given that the researcher plays no role in creating the DAA, drafters must consider other elements that will create a stronger contractual bond, such as a shared set of values, and a sense that the research being embarked upon is a shared endeavour. Harmonization can help to reinforce a set of common goals for researchers, thus facilitating a sense that they are committing to a set of mutually agreed upon, robust *community* norms and principles

**Fig. 1:**
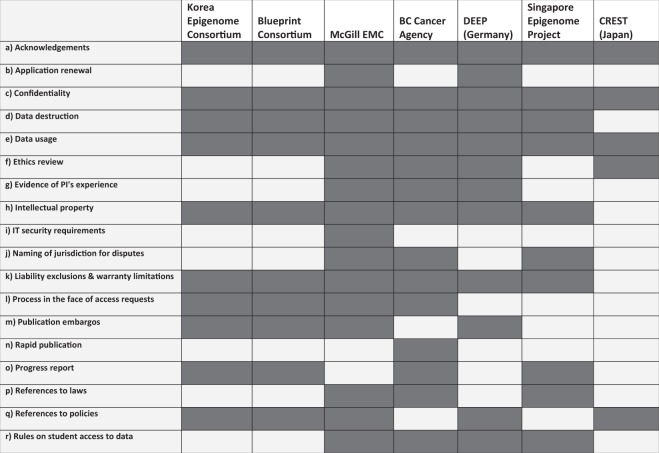
Terms and Conditions identified in IHEC DAAs.

.

In order to assess the types of clauses that were included in IHEC’s DAAs, categories were defined very broadly. While some terms used similar language across all agreements, in other cases there was significant variation. Clauses were counted in our table as being included if they touched on a given category; for instance, DAAs were counted as providing rules on access to students whether this was in the context of prohibition or permission. Agreements were counted as touching on IT security requirements whether they simply required that a policy be in place, or provided a policy themselves. The goal in classifying the clauses was to assess which types of concerns were prevalent across the agreements, after which an assessment was made of how the content of these clauses fit with international ethical and legal norms.

Some types of terms were ubiquitous. All seven DAAs considered who should be able to use the data, and to what end. In keeping with the legal and ethical importance of protecting privacy, all seven DAAs required that the confidentiality of the data be maintained (Fig. 1, c). Four DAAs addressed whether or not students may access the data and under what terms, ranging from requesting a list of all students who will work on the project to the requirement that PhD students list their supervisors as co-applicants (Fig. 1, r). Three DAAs also included provisions requiring evidence of research and data-handling competence on the part of the applicant researcher (Fig. 1, g), demonstrated by way of either a list of relevant previous publications or a more general description of research interests and experience. An approach that allows for both options may be preferable in order to include researchers at the start of their careers.

The requirement that the researchers provide the institution with a description of the planned research, with the understanding that the data were being provided solely for the purposes of undertaking the described research, was present in all agreements (Fig. 1, e). These clauses are in place to help ensure that data are used only for research purposes that are not contradictory to the interests of and consent given by research participants. It is not clear, however, if the project description was also used by DACs to screen for scientific merit. We note that such screening is inconsistently and ambiguously applied across other databases as well; a 2014 review of biobanking DAAs showed that only half indicated whether or not applications would be screened for scientific merit^15^. Four out of seven agreements additionally required ethics approval by a Research Ethics Board/Institutional Review Board, although two of these required approval only if an ethics review is mandatory in the region where the research will take place (Fig. 1, f).

In four agreements, the data provider required a research report upon completion of the user’s project (Fig. 1, o). Additionally, clauses that require researchers to acknowledge in any publications the provenance of the data, were present in all seven agreements (Fig. 1, a). This requirement is in line with international data sharing guidelines, which state that “[t]he contributions and interests of the large-scale data providers should be recognized and respected by the users of the data”^16^. This is done in part to incentivize data sharing by rewarding data providers who share their data^11^, and as a way to enrich, promote, protect, and sustain their resources without any extra cost to the researcher^4^. Other common clauses, however, may more negatively impact data sharing. Publication embargos – periods where publication on data analysis is restricted to the data providers – are common in DAAs, despite concerns that their overuse may present an impediment to the principles of open science^17^. Indeed, while four of the DAAs we reviewed included provisions that the project be given the first opportunity to publish global analyses of the datasets, only one DAA requested that researchers coordinate with the data providers to make their results available as quickly and widely as possible.

## Recommendations

Based on literature on data sharing best practices, as well as our own experience, we assessed those clauses where there was great intra-consortium variation in order to generate recommendations of the types of clauses to include or exclude in harmonized and streamlined DAAs. We attempted to balance the need for participant protection and data sharing in a manner that reflects provisions of ethical guidelines and professional norms such as the Toronto Statement^18^ and the policy work of the Global Alliance for Genomics & Health (GA4GH)^19^, as well as literature on the ethical concerns surrounding data sharing^20^. All of these highlight the importance of open data sharing for health research that is conducive to the public good. As we move increasingly toward a climate of open science, to reap the full benefits of genomic research, data access terms and conditions that detract from these principles are considered justifiable only insofar as they are necessary to protect the interests of research participants^1^.

## Clauses to Include

Certain types of clauses have a straightforward positive impact on either data sharing or participant protection, yet were not included consistently across DAAs. Clauses requiring that the researcher adhere to up-to-date IT security best practices, such as logging and auditing data access and encryption of devices where data is stored; clauses requiring an application renewal process after a specific timeframe has passed; clauses requesting evidence of experience from PIs; and clauses that encouraged rapid publication were only included in four or fewer DAAs. Others – such as clauses setting the terms for data usage (e.g. requiring that the data be used only for approved projects), requirements that data providers must be acknowledged in resulting publications, and provisions requiring the destruction of data at the end of the project – were already included in most or all of the agreements.

## Clauses that Require Caution

A second category of clauses are useful in some contexts, but have benefits that should be weighed against other potentially detrimental effects. Clauses requiring ethics approval are acceptable, but should be commensurate to the sensitivity of the data provided so as not to impede data access when it is not required for participant protection. Clauses that outline publication embargo periods (not data retention) are also acceptable *if* the embargo period is not overly long. In contrast with data retention policies, embargos still enable other researchers to view the data. As such, these embargos are less concerning than data retention policies, where the data provider does not make the data available until their own initial research is completed. However, if they allow too great a period to elapse before allowing external researchers to publish, they can present a serious impediment to open science. A formulation that encourages the shortest possible time frame will be beneficial, such as: “*You agree to a moratorium on publishing global analyses of the dataset until Data Producers have published their own global analysis or […] months have passed from the time the data is deposited*, *whichever occurs first*.” Finally, references to policies or guidelines should be limited, with the understanding that researchers may not take the time to fully read appendices or referenced documents; any important policy points should be pulled into the text of the DAA itself.

Of these clauses for which we recommend caution, two particularly stood out both for their inconsistent implementation across the analyzed DAAs and for the length and complexity with which they were drafted.

### Limitation of liability

First, several DAAs contained limitation of liability clauses that were of questionable benefit for the protection of data providers; in trying to cover all possible circumstances, either through the use of blanket statements or a long list of hypothetical scenarios, the clauses may be too broad to effectively enforce^21^. For instance, some DAAs included extensive clauses limiting or excluding data producer liability. However, ambiguity or unnecessary breadth in clauses to limit liability, particularly when they make specific references to domestic laws, can lead courts of law or arbitrators to interpret them as null, or to rule against the interests of the person invoking them^22^. A better approach would simply be to address the main areas of concern in the context of data sharing in plain language. Examples of these categories might include liability for integrity of data content; liability for interruptions to data access; liability for how the data is interpreted; and liability for how researchers use the data (e.g. patent infringements).

### Intellectual property

Secondly, some DAAs included extensive provisions aimed at protecting intellectual property (IP). Excessive IP encumbrance is a hindrance to the advancement of science and to broad access to treatment, and can lead to costly litigation^23^. Chokshi *et al*. propose two principles that should underpin IP considerations in large-scale genomic research collaborations: “(1) impediments to innovation in research processes should be minimized, and (2) the fruits of research – eventual products that result from scientific discoveries – should be made as widely accessible as possible, particularly to the people who need them most”^24^. Although the non-patentability of primary data is now recognized by patent laws in many countries, there is still some institutional resistance to the idea that raw data collected in publicly funded projects should not be held in silos by way of property rights. There exist a number of legal routes to fulfilling Chokshi’s proposed principles, and these are discussed at great length in existing literature^24^. For now, we argue that IP clauses should focus on allowing the data to be used as widely as possible, while still protecting the IP rights of researchers for downstream discoveries when it can be shown that treatments could not otherwise become available.

## Clauses to Exclude: Clauses referring to Specific Laws and Jurisdictions

A major issue in harmonizing DAAs is that of ensuring comprehensibility, consistency, and enforceability across distinct legal regimes^25^. The DAAs we examined made reference to national laws (Fig. 1, p) and responses to Freedom of Information requests (Fig. 1, l). While the importance of protecting individuals’ privacy and confidentiality are well recognized, approaches to this privacy protection can differ from one jurisdiction to the next. The increasingly international nature of data sharing challenges DACs to maintain the level of protection promised to participants without real power to enforce those laws on researchers in other jurisdictions. References to specific local or national laws can discourage researchers from applying to use the data, as they are unlikely to be sufficiently familiar with these laws to guarantee compliance. Instead, it may be more effective for DAAs to provide a clear and concise set of terms and conditions regarding data handling, security, and re-identification that meet or exceed the protections promised to participants in both their consent forms and in local laws.

## Conclusions

A controlled data sharing practice, wherein each individual academic institution focuses on protecting its own perceived proprietary interests in data instead of viewing restrictions on open access as exceptional and only justified to protect the privacy interest of research participants, is detrimental to the interest of both researchers and research participants. Without some degree of simplification and harmonization, the “controlled access” edifice, built to facilitate ethical scientific research, threatens to become instead a source of frustration for the research community.

Harmonization of DAAs will not be the solution for all types of research, nor will it be the lone solution even in the context of large consortia such as IHEC. Research with vulnerable participants, such as pediatric research, research with adults who are incapable of providing consent, and research targeted toward marginalized communities, for instance, are subject to different considerations and regulations^26^. Moreover, even within contexts where harmonization is appropriate, DAAs and DAC procedures cannot act as the sole bulwark to protect participants and facilitate open science. The approach must be complemented by appropriate regional and/or national privacy legislation and the use of up to date information technology security norms, programs and tools (ex. firewalls, encryption protocols, password protected access, antivirus protection, etc.).

Although this commentary raises several points of discussion, a broad community consensus will be required to accomplish changes in DAC’s practices. Fortunately, the issue is beginning to attract further attention; a number of initiatives, including GA4GH’s Data Use Ontology (DUO) and Automated Data Access Matrix (ADA-M) projects, as well as the Broad Institute’s Data Use Oversight System (DUOS), are investigating mechanisms for regulating data access via standardized language for data use restrictions allowing for more automation of data sharing processes. In addition to the intra consortium harmonization attempt reported here for IHEC, the EU Standards for Personalised Medicine (EU-STANDS4PM) project is currently evaluating the possibility of inter consortia harmonization of DAAs across multiple projects funded under the H2020 Programme. Because of the myriad stakeholders involved in the data-sharing process, an international meeting of administrators of DACs of major genomics projects, and related sciences across the globe, is being planned in order to examine the challenges of DAA harmonization; to develop quality improvement strategies for the review process; and more broadly to discuss their respective experiences in providing access to controlled data.

There are preliminary steps, outlined here, which can facilitate greater harmonization of DAAs. The next step will be to engage DACs from across different cultures and contexts in order to consider how to best navigate toward the development of shared norms that balance the interests of participant protection and open science effectively. Meanwhile, greater attention to the design and use of DAAs can be a powerful tool in fostering better collaboration between researchers and data providers, encouraging compliance with shared principles, generating more efficient and effective knowledge translation, and ultimately facilitating large-scale international data sharing.
